# Point-of-care Ultrasound Evaluation of Tibial Avulsion Fractures

**DOI:** 10.7759/cureus.2677

**Published:** 2018-05-23

**Authors:** Josie Acuna, Elaine Situ-LaCasse, Robert P Jamplis, Richard Amini, Srikar Adhikari

**Affiliations:** 1 Department of Emergency Medicine, University of Arizona, Tucson, USA; 2 Department of Emergency Medicine, University of Arizona, Tucson, AZ; 3 College of Medicine, University of Arizona, Tucson, USA

**Keywords:** ultrasound, emergency medicine, point-of-care ultrasound, orthopedic trauma

## Abstract

It can be difficult to diagnose a tibial avulsion fracture based on physical examination alone as findings are often non-specific. Emergency physicians will usually opt for radiography as their initial imaging modality, which has several disadvantages in evaluating tibial avulsion fractures. The objective of this case series is to describe the utility of point-of-care ultrasound (POCUS) in the evaluation of tibial avulsion injuries.

A 15-year-old male presented to the emergency department (ED) after sustaining a left knee injury while playing soccer. The clinician had a high suspicion for patellar tendon involvement. A POCUS exam revealed a cortical irregularity and interruption of the left proximal tibia. The patellar tendon was found attached to an avulsed bony portion. Findings were consistent with a tibial tuberosity avulsion fracture. The patient was admitted and scheduled for surgery the following day.

Our second case is a 31-year-old male who presented to the ED with a complaint of left knee pain after a 10-foot fall from a ladder. A POCUS exam revealed a bony avulsion over the anterior tibia that was not noted on the initial radiography read by radiology. His patellar tendon showed no evidence of rupture. This led to prompt consultation with orthopedics who evaluated the patient in the ED. Radiographs were reviewed again and it appeared that there was a missed anterior tibial spine fracture. The patient was placed in a knee immobilizer and discharged with instructions to follow up with orthopedics for outpatient surgery.

The use of POCUS in the evaluation of these patients led to prompt diagnosis of tibial avulsion injuries, which led to early consultation and appropriate patient management. POCUS allows for expedited diagnosis and appropriate management of patients with tibial avulsion injuries.

## Introduction

Emergency physicians are often the first to encounter patients who sustain musculoskeletal injuries. A correct diagnosis of tibial avulsion injuries is necessary for establishing the appropriate treatment and follow up for the patient. It can be difficult to distinguish between a muscle strain and an avulsion fracture based on physical exam alone, especially when a patient arrives acutely in the emergency department (ED). Localized tenderness to palpation and loss of function, as with flexion or extension of a joint, is often not specific. If a clinician finds that imaging is indicated, they will usually opt for radiography as their initial imaging modality. Unfortunately, radiography has several disadvantages in the evaluation of a tibial avulsion fracture. To make the diagnosis, visualization of the dislocation or fragmentation of the bony protuberance on imaging is necessary. This can be difficult to identify if the avulsed bony fragment is small or if the ossification of an avulsed apophysis is not complete, as often seen with younger patients. Ultrasonography has been found to be an appropriate diagnostic tool for such cases. Its advantages include the ability for clinicians to perform dynamic studies and allow for evaluation of tendon injury associated with an avulsed portion. It also minimizes the radiation a patient will receive as opposed to radiography.

This case series reports two types of cases of tibial avulsion fractures diagnosed in the ED with the utilization of point-of-care ultrasound (POCUS).

## Case presentation

Case 1

A 15-year-old, otherwise healthy male, presented to our ED for evaluation of a left knee injury. The patient stated that just prior to arrival he was playing soccer when he felt a "pop" just below his left knee as he was about to kick the ball. This was followed by immediate pain. The patient had been unable to bear weight on that knee ever since. In the ED, the patient reported his pain as an 8/10 in severity, worse with movement. Physical exam demonstrated an edematous left knee anteriorly. There was significant tenderness to palpation just inferior of the left patella. The patient’s knee was held in minimal flexion, without the ability to fully extend. He was neurovascularly intact throughout.

Radiographs were obtained of the left knee, tibia, and fibula. Radiographs demonstrated a fracture of the left tibial tubercle. The visualized osseous structures were otherwise in anatomic alignment and the joint spaces were preserved. There was no significant left knee joint effusion identified.

**Figure 1:**
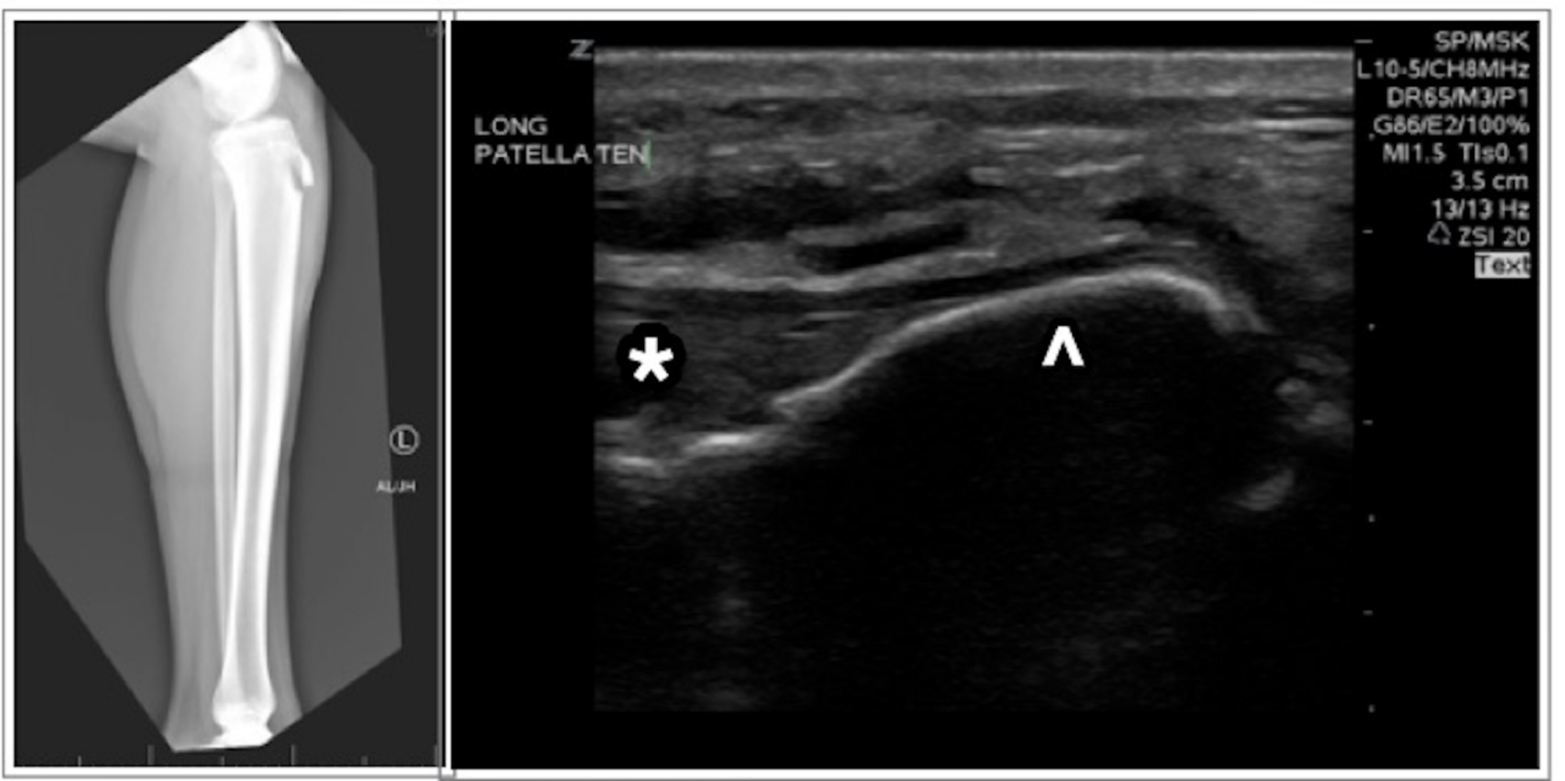
Radiograph demonstrating fracture of the left tibial tubercle (left); ultrasound of the knee (right) demonstrating avulsed tibial tuberosity (arrowhead) with attached patellar tendon (*)

Given the location of the fracture and the patient’s inability to extend at the knee, the clinician had a high suspicion for patellar tendon involvement. A POCUS exam was performed by the treating physician using high-resolution ultrasound (10-5 MHz linear array transducer). The ultrasound revealed a cortical irregularity and interruption at the left proximal tibia, consistent with a displacement of the tibial tuberosity. The patellar tendon was confirmed to still be attached to the avulsed portion (Figure 1).

The patient was admitted and scheduled for surgery the following day to correct the left tibial avulsion fracture. Open reduction and internal fixation of the left tibial tubercle was performed successfully.

Case 2

A 31-year old man presented to the ED with a complaint of left knee pain after a 10-foot fall from a ladder three days prior. On exam, the patient was unable to fully extend the left knee. He had a marked effusion without warmth or erythema. The patient was neurovascularly intact throughout. Radiographs of the left knee were performed which demonstrated cortical irregularity of the medial tibial plateau consistent with medial tibial plateau fracture. Given the extent of the edema and limitation in range of motion of the knee, the decision to perform a POCUS was made to assess for further injury.

**Figure 2:**
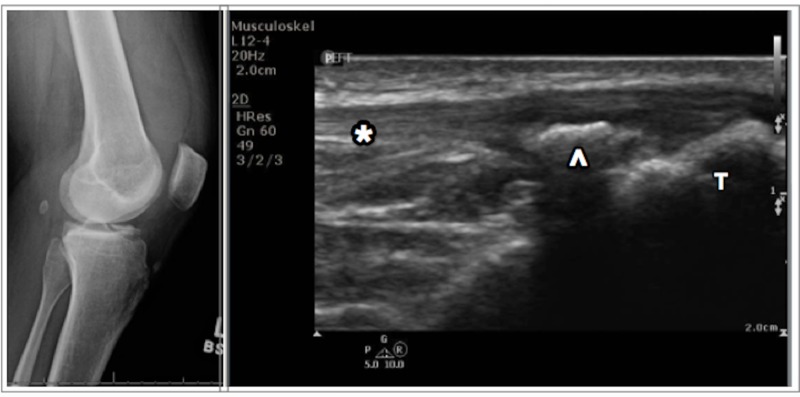
Radiograph of the left knee (left); ultrasound of the knee (right) demonstrating the tibia (T) with an avulsed portion of the anterior tibial spine (arrowhead), and an overlying intact patellar tendon (*)

The ultrasound examination was performed by the treating emergency physician using a 12-4 MHz linear array transducer. The examination revealed significant hemarthrosis of the left knee. The patellar tendon showed no evidence of rupture. A bony avulsion over the anterior tibial spine was visualized that had not been noted on initial radiography in the ED (Figure 2).

The patient was seen by orthopedics in the ED. After evaluation of the patient and discussion of the ultrasound findings, the radiographs were reviewed once again and it appeared that there was likely a missed anterior tibial spine fracture. The patient was placed in a knee immobilizer and discharged with strict instructions to follow up with orthopedics for outpatient surgery.

## Discussion

In the first case presented, the patient sustained an avulsion fracture of the tibial tuberosity. Avulsion fractures of the tibial tuberosity are rather uncommon, accounting for less than 1% of all physeal injuries. Their estimated incidence ranges from 0.25 to 2.7 cases per year [1-3] The tibial tuberosity gives attachment to the patellar ligament, which attaches superiorly to the patella from where the suprapatellar ligament forms the distal tendon of the quadriceps femoris muscles. There are two typical mechanisms of injury. The first is a powerful quadriceps contraction during knee extension. The second is a rapid passive flexion of the knee against the contracting quadriceps. These types of avulsion fractures typically occur in athletic males, usually during adolescence as they are reaching skeletal maturity. Osgood-Schlatter disease has often been indicated in literature as a predisposing factor for this type of fracture [4-5].

Another example of a tibial avulsion fracture that might be encountered in the acute care setting was demonstrated in the second case. This avulsion occurs at the anterior tibial spine, a site of attachment for the anterior cruciate ligament (ACL). These fractures are characterized by disruption of the bone underlying the tibial insertion site of the ACL as it pulls away from the proximal tibia. Similar to avulsions of the tibial tuberosity, they commonly occur in skeletally immature adolescents, due to the inelastic nature of the incompletely ossified tibial spine relative to the fibers of the ACL [6-7]. Radiography may miss these when there is an incompletely ossified tibial spine.

While the gold standard for diagnosing intra-articular knee pathology is arthroscopy, initial imaging is almost always needed prior to a patient being taken for such a procedure. Magnetic resonance imaging (MRI) is a non-invasive method with a high sensitivity and specificity for intra-articular pathology [8-9]. However, while MRI is highly sensitive for detecting such injuries, it is not always feasible or even possible to obtain an MRI in the ED setting. The initial imaging modality of choice for most knee injuries presenting to the ED is radiography. Unfortunately, radiography has several disadvantages in the evaluation of traumatic knee injuries, such as avulsion fractures. In a study by Lazovic et al., 243 patients with clinically suspected avulsion injuries of the lower extremity were reviewed. In each patient, both radiography and an ultrasound examination were performed. In 80 cases, the diagnosis was confirmed by radiography and in 97 by ultrasonography. This study emphasized the utility of ultrasound for avulsion injuries, specifically in the lower extremity. Authors concluded that not only is ultrasound a useful modality to detect apophyseal injuries, but it is also actually far superior to radiography in some cases [10].

**Figure 3:**
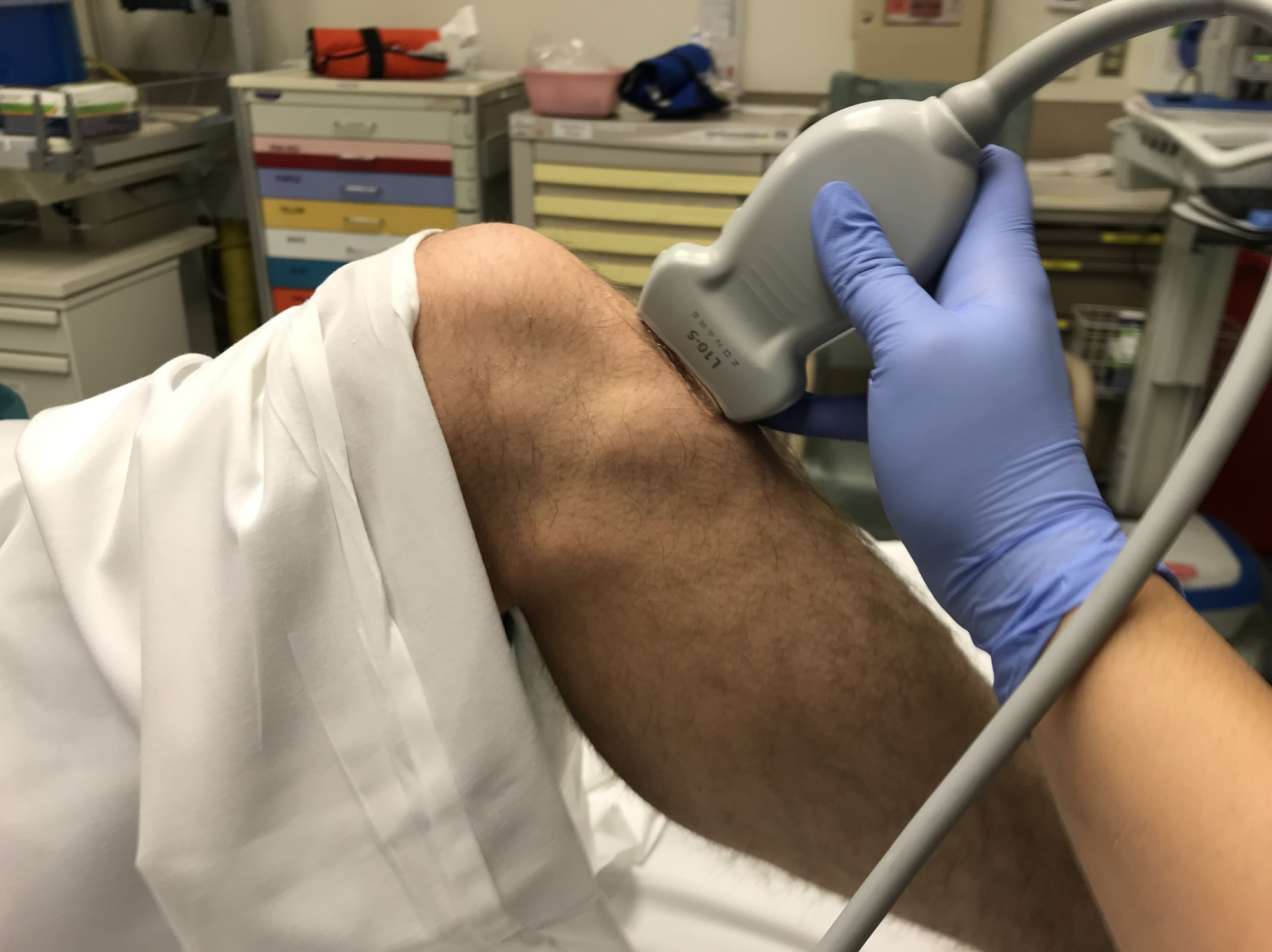
Patient and probe positioning

When performing this ultrasound, the patient is scanned using a high-frequency linear array transducer. Comparison with contralateral normal side and dynamic imaging are recommended. The knee is scanned anteriorly just below the patella to obtain visualization of the proximal tibia (Figure 3). Scanning through the proximal tibia in long axis allows for visualization of any avulsions or fragmentation of the bony protuberance. In this view, the operator can evaluate for infrapatellar tendon injury as well. A full tendon rupture is characterized by complete disruption of the normal pattern of parallel fibers in the long axis view, often with retracted tendon ends. Additional sonographic findings include hematoma formation at the site of rupture, posterior acoustic shadowing at the margins of the rupture, and adjacent hypoechoic tendinosis [11].

## Conclusions

These cases illustrate the use of point-of-care musculoskeletal ultrasonography in the evaluation of patients with suspected tibial avulsion fractures, especially when the physical examination is limited or equivocal. POCUS can potentially avoid misdiagnosis and direct appropriate care and follow-up for these patients.
